# Water Sorption and Solubility of Flowable Giomers

**DOI:** 10.3390/ma14092399

**Published:** 2021-05-05

**Authors:** Mara Elena Rusnac, Doina Prodan, Stanca Cuc, Ioan Petean, Cristina Prejmerean, Cristina Gasparik, Diana Dudea, Marioara Moldovan

**Affiliations:** 1 Department of Conservative Odontology, Iuliu Hatieganu University of Medicine and Pharmacy, 33 Motilor Street, 400001 Cluj-Napoca, Romania; rusnac.mara@umfcluj.ro; 2 Department of Polymer Composites, Institute of Chemistry Raluca Ripan, Babes-Bolyai University, 30 Fantanele Str., 400294 Cluj-Napoca, Romania; stanca.cuc@ubbcluj.ro (S.C.); cristina.prejmerean@ubbcluj.ro (C.P.); marioara.moldovan@ubbcluj.ro (M.M.); 3 Faculty of Chemistry and Chemical Engineering, University Babes-Bolyai, 11 Arany János Str., 400028 Cluj-Napoca, Romania; ioan.petean@ubbcluj.ro; 4 Department of Prosthetic Dentistry and Dental Materials, Iuliu Hatieganu University of Medicine and Pharmacy, 32 Clinicilor Street, 400006 Cluj-Napoca, Romania; gasparik.cristina@umfcluj.ro (C.G.); ddudea@umfcluj.ro (D.D.)

**Keywords:** flow giomers, sorption, solubility, morphology, SEM, AFM

## Abstract

The objective of this study is the characterization of a novel experimental flowable giomer (G) regarding water sorption, water solubility, and the microstructural characteristics, in comparison to three commercial giomers: Beautifil flow Plus X F00 (B-F00), Beautifil flow F02 (B-F02) and Beautifil flow Plus X F03 (B-F03), Shofu, Kyoto, Japan. Methods: Water sorption/solubility was performed by weighing the specimens before and after water immersion for 1, 2, 3, 14, 21 and 30 days. Data analysis was carried out with the software Origin2019b Graphing & Analysis using the ANOVA test and the Tukey test for post hoc comparison of the groups of materials. The microstructural analyses were done with a scanning electron microscope (SEM) and an atomic force microscope (AFM). The results showed significant differences between the tested materials (*p* < 0.05). For sorption, the Tukey test indicated differences between all four sample groups, except between B-F02 and B-F03, which exhibited no differences in any of the investigation days. The Tukey test also showed significant differences regarding solubility between all sample groups in the 30-day interval. SEM images and roughness showed that after 30 days of immersion in water, the experimental giomer G had the roughest surface.

## 1. Introduction

Giomers are hybrid materials, having a resin matrix and pre-reacted glass filler (PRG) and controlled fluoride releasing properties. They comprise the easy handling and esthetic properties of composite resin materials and the carious protection thru fluoride release offered by glass-ionomers [1]. The PRG filler is the result of an acid–base chemical reaction between a fluoroalumino-silicate glass and polyalkenoic acid (PAA) in the presence of water, leading to a glass-ionomer in a stable form (“wet silicon hydrogel”) [2].

Commercially, giomers have been introduced on the market by Shofu Dental (Japan) which provided different consistencies, adapted for various clinical situations: conventional (indicated for reconstruction of tooth anatomy when important portions of the dental structure are missing) and flowable giomers, with more specific indications, according to their individual consistencies: high stress areas, like class V restorations, or marginal ridges, cavity liners, and small restorations [3,4,5,6].

In general, a flowable dental restoration material has lower mechanical properties and higher fluidity when compared to a conventional one, however, the variability in their consistency was aimed for to answer different clinical goals. A zero-flow material (Beautifil Flow Plus X F00, B-F00) was developed as a stable flowable giomer that can withstand the restoration of the occlusal anatomy, marginal ridges and all anatomical details of all filling classes, whereas a moderately flowable one (Beautifil Flow Plus X F03, B-F03) was indicated for class V restorations or as a cavity liner [7]. A low-flow material (Beautifil Flow F02, B-F02) and a highly fluid one (Beautifil Flow F10) were indicated for small or superficial restorations (class I to III and class V) and also as cavity liners [8]. Resin-based flowable materials have the advantage of high flexibility, ability to reduce the setting contraction and obtain a superior sealing of the marginal interface, along with a superior bond to both enamel and dentin [9]. Commercial products were improved over time, reaching higher performances regarding different properties, including wet exposure behavior. B-F02 was an early generation of a flowable giomer material for which a lot of experience exists. The newest additions in the class of flowable giomers are the B-F03 and B-F00. The material selection was aimed to allow for a better understanding of the experimental material G properties.

However, to our knowledge, very few references are available in the literature about flowable giomers [3,9,10].

Water sorption is a diffusion-controlled process, leading to an increase in volume and inflation of the material and over time to hydrolytic degradation of the material and lower mechanical properties. Sorption and solubility can be influenced by the matrix composition, degree of conversion, type, shape, size of the filler and filler percentage. Sorption and solubility can have a big impact on the mechanical properties, structural integrity, dimensional stability and color of resin-based materials. Because many dental treatments have esthetic motivation, color changes at the surface or margins of a restoration are unacceptable. Optical properties of flowable giomers were found to be also correlated to the filler amount in each material. Overall, flowable giomers were determined to be highly translucent and having the low masking capacity of a dyschromic substrate [10]. Hydric degradation of a restoration material leads to a rougher surface, which in turn favors significant plaque deposits. Material disintegration and dissolution are the result of the penetration of oral fluids through surface micro-fissures. Solubility will lead to a decrease in weight per unit of volume because of leaching certain components as a result of exposure to oral fluids. This complex process is influenced by the quantity of the residual monomer as consequence of the polymerization process, elutable components, type of solvent and the composition of the leached material. It is therefore necessary to thoroughly investigate the sorption and solubility of a resin-based material, given the influence these can have on the stability and performance of the restoration over time [4,5,11].

In this study, it was aimed to test clinically relevant characteristics of the selected materials (a flowable experimental giomer and three commercial giomers): water sorption, water solubility and to provide microstructural analyses of the sample surfaces before and after immersion in liquid.

The objective of this study is to analyze an experimental flowable giomer, prepared in the laboratory of Babes Bolyai University UBB-ICCRR regarding sorption and solubility in distilled water; changes in roughness and microstructural analyses of the surface, before and after the immersion in distilled water, were also examined. The comparison of the experimental giomer material was done against three variants of commercial giomers: Beautifil Flow Plus X F00, Beautifil Flow F02, Beautiful Flow Plus X F03 (Shofu Dental, Japan), all in A2 color.

The null hypothesis was that no statistically significant differences could be found among the experimental and commercial materials.

## 2. Materials and Methods

### 2.1. Studied Materials

#### Experimental Light-Curing Giomer Fabrication

The experimental giomer was obtained as mono-pastes by mixing the resin with the mixture of hybrid fillers.

The organic matrix is composed of Bis-GMA: analogue (93% 2,2-Bis[*p*-(2-hydroxy-3-methacryloyloxypropoxy)-phenyl]-propane monomer and 7% dimer) as the basic monomer, triethylene glycol dimethacrylate (TEGDMA) as the diluting monomer and photochemical initiation system components: 0.5% camphorquinone (CQ) as the photosensitizer, 1% dimethylaminoethyl-methacrylate (DMAEM) as the accelerator. Bis-GMA was obtained at the Babeș-Bolyai University, Raluca Ripan Institute for Research in Chemistry, (Cluj-Napoca, Romania) [12]. The other components were purchased from Sigma Aldrich Chemical Co. (Taufkirchen, Germany).

FHAP (fluorohydroxy apatite), Exp-Glass, and S-PRG were also synthesized in the laboratory of UBB-ICCRR. The experimental glass (Exp-Glass) powder is based on barium fluoro-alumino-boro-silicate glass silanized with 3-methacryloyloxypropyl-1-trimethoxy-silane (A-174 silane) from Sigma Aldrich Chemical Co. FHAP (fluorohydroxy apatite) is composed of needle-shaped nanometric particles, 15–160 nm in length and 10 nm in width. The detailed descriptions of obtaining the powders are presented by Prejmerean et al. and Burtea et al. [6,13].

The materials selected for this experiment were: an experimental flowable giomer and three commercial materials: Beautifil Flow Plus X F00, Beautifil Flow F02 and Beautifil Flow Plus X F03 from Shofu, Kyoto, Japan. The composition of all selected materials is listed in Table 1.

### 2.2. Water Sorption and Water Solubility

For testing the water sorption and water solubility, six samples were fabricated for each material (7.5 mm radius and 1 mm thickness) according to ISO 4049 [14] (N = 6). The light curing was performed for 20 s with an LED.E (GuilinWoodpecker Medical Instruments Co., Guangxi, China), having wavelengths in the range of 470 nm and an intensity of 950 mW/cm^2^. No surface finishing treatment was performed. The samples were stored for 24 h in a desiccator. After this, they were weighed multiple times until a constant mass was determined, and this was considered to be the initial mass (m1). The thickness and diameter of each sample were measured, with a digital precision measurer, in three distinct areas.

The volume of each sample was calculated with the following formula: V = πr^2^h [mm^3^], with r representing the medium radius, h representing the medium thickness, and V representing the volume.

All samples were then stored in 30 mL of distilled water, in individual glass containers, for a period of 30 days, in a thermostatic bath, at 37 °C (±2). After 24 h, the samples were removed from the containers, dried with filter paper and then in air for 15 s. Each sample was weighed three times, a minute after being removed from the container and dried, and this value is called m2. The next step was to maintain the samples in a desiccator, to obtain a constant mass, m3. Following the above-described process, the weighing was performed on day 2, 3, 7, 14, 21, and 30.

The results for the sorption (Sp) of water and the solubility (Sl) of the samples are expressed in (μg/mm^3^) and were calculated with the following Equations (1) and (2) [14]:

Sp = (m2 − m3/V)(1)

Sl = (m1 − m3/V)(2)

where m1 represents the initial mass before immersion in water; m2 represents the mass after immersion at a moment in time; m3 represents the final mass after the sample was dried in the desiccator, and V is the volume of the samples.

### 2.3. Microstructural Analysis of Giomer Surfaces by Scanning Electron Microscopy (SEM) and Atomic Force Microscopy (AFM)

All the selected samples used in the water sorption test were investigated by scanning electron microscopy (SEM) and atomic force microscopy (AFM) to characterize the initial state of the material surface.

#### 2.3.1. Scanning Electron Microscopy (SEM)

The surface structure of the representative sample for each investigated giomer material, before and after storage in distilled water after a 30-day period, was performed with a scanning electron microscope (SEM-Inspect S, FEI) at a magnification of ×5000.

#### 2.3.2. Atomic Force Microscopy (AFM)

The AFM investigation was performed on a JEOL JSPM 4210 Scanning Probe Microscope, Tokyo, Japan, in tapping mode. The used cantilevers are NSC 15 type produced by MikroMasch, Sofia, Bulgaria. The cantilever characteristics are: resonant frequency 325 kHz and force constant 40 N/m. The topographic images were scanned at an area of 5 µm × 5 µm at a scan rate in the range of 1 to 1.5 Hz. All images were processed in the standard manner using the Jeol Win SPM 2.0 Processing software which allows to measure surface parameters such as the Ra and Rq roughness. The average values were determined using at least five images obtained on different macroscopic areas on the sample surface.

### 2.4. Statistical Analysis

The data were analyzed with the ANOVA and Tukey tests for post hoc comparison between the sample groups. The level of significance is α = 0.05, and the analyses were performed with the Origin2019b Graphing & Analysis software (OriginLab, Northampton, MA, USA).

## 3. Results

### 3.1. Water Sorption and Water Solubility

#### 3.1.1. Water Sorption

**Figure 1:**
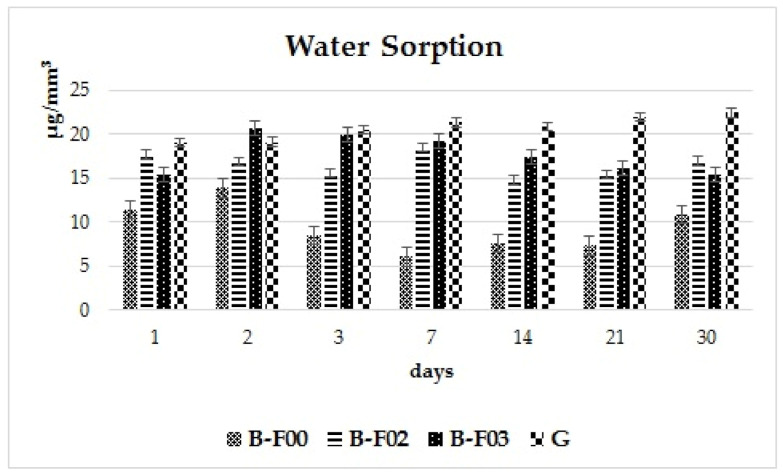
Water sorption of the investigated materials.

Mean values of water sorption/day for each of the investigated materials are presented in Figure 1. On the first day, the maximum value was registered for G, the experimental giomer (19.08 μg/mm^3^) and on the second day, for B-F03 (20.64 μg/mm^3^). The highest water sorption values were registered for the experimental giomer material G on day 3, 7, 14, 21, 30 of the evaluation period, increasing slowly (22.44 μg/mm^3^, day 30). The lowest water sorption values for each of the investigation days were registered for material B-F00, with the second day registering an increase compared to the first day (13.87 μg/mm^3^ compared to 11.38 μg/mm^3^) and decreased at the end of the period to 10.85 μg/mm^3^.

For the giomer material B-F02, the water sorption during the first day was 17.68 μg/mm^3^. In the following days, the values decreased and, on the 7th day, increased to 18.5 μg/mm^3^, followed again by a decrease on the 14th day and a discrete increase by the 21st. At the end of the investigation period, the water sorption reached values up to 17 μg/mm^3^, close to the registered values of the 1st day.

Material B-F03 registered water sorption values of 15.46 μg/mm^3^, followed by an increase to 20.64 μg/mm^3^ on the second day and then decreasing slowly until the last day of the investigation period. By the 30th day, the registered value was 15.41 μg/mm^3^, nearly identical to that of the first day of investigation.

**Figure 2:**
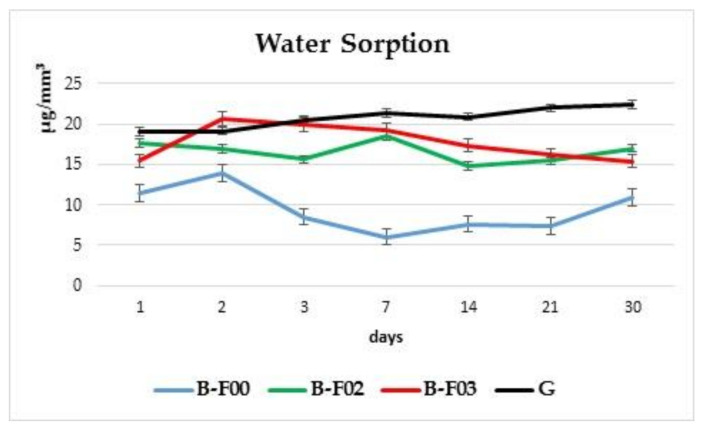
Water sorption of each individual material over the entire investigation period.

Figure 2 was added for a better visualization of the behavior of each individual material over the entire investigation period. The experimental giomer G exhibits a maximum of water sorption on the last day of the experiment (22.44 μg/mm^3^). However, the water sorption increase from the first until the last day of immersion was not significant (3.36 μg/mm^3^).

Giomer B-F00 reaches the greatest sorption value on the 2nd day (13.88 μg/mm^3^), decreasing afterwards and slightly increasing during the last two weeks of the experiment (registering a difference of 0.53 μg/mm^3^, between the first and last day).

Giomer B-F02 displayed a slight decrease after the first day of immersion and reaches a maximum value on the 7th day (18.50 μg/mm^3^), followed by another decrease. On the last day, the registered value was close to the one from the first investigation day (the difference between the first and last day is 0.68 μg/mm^3^).

Giomer B-F03 has the highest value of water sorption on the 2nd day (20.64 μg/mm^3^), followed by a decrease over the entire 30-day period (the difference registered between the first and last day is 0.06 μg/mm^3^).

This analysis of the behavior displayed by giomer materials, when exposed to a wet environment, shows that for the commercial giomers, the water sorption value on the last day are close to the value registered on the first day. The highest overall water sorption value, without significant increases or decreases over the investigation period, was registered for the experimental giomer.

#### 3.1.2. Water Solubility

**Figure 3:**
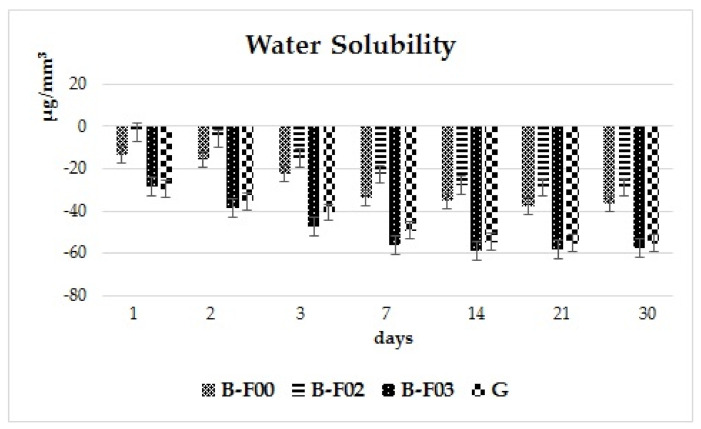
Water solubility of the investigated materials.

**Figure 4:**
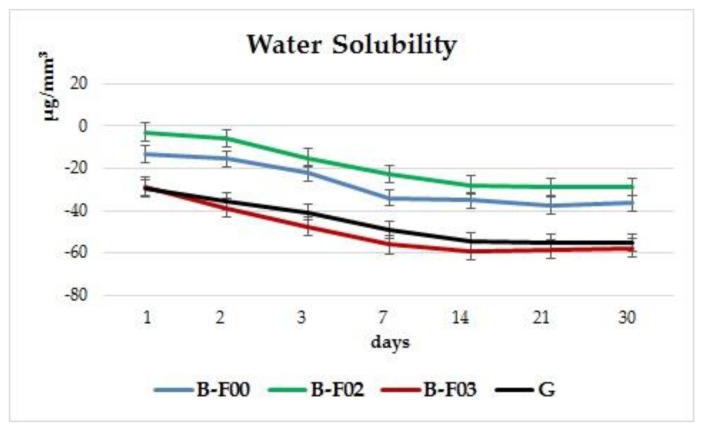
Water solubility of each individual material over the entire investigation period.

In Figure 3 it is shown that all the investigated materials have negative values for solubility and for a better understanding of the materials behavior Figure 4 was added. After the first day, the highest mean values registered for the solubility parameter were for the experimental giomer G (−29.63 μg/mm^3^); on day 14, a value of −54.73 μg/mm^3^ was registered, and by the 30th day, −55.33 μg/mm^3^.

Slightly lower mean values were obtained for B-F03: after 24 h, −28.70 μg/mm^3^, after 14 days, −59.20 μg/mm^3^, and −57.68 μg/mm^3^ by day 30, the end of the investigation period.

Giomer B-F02 registered the lowest value for the solubility parameter: after 24 h, the mean value was −2.81 μg/mm^3^, after 14 days, −27.86 μg/mm^3^, and −28.75 μg/mm^3^ after 30 days, the end of the investigation period.

Giomer B-F00 registered mean values of: −13.42 μg/mm^3^ on the first day, −35.16 μg/mm^3^ on day 14, and −36.49 μg/mm^3^ at the end of the investigation period.

In Figure 4 the water solubility is displayed. The experimental giomer G registers values close to the commercial giomer B-F03 for this parameter. The differences between the first and the last day of investigation are 25.7 μg/mm^3^ (for G) and 28.98 μg/mm^3^ (for B-F03), respectively.

Giomers B-F02 and B-F00 register differences between the first and the last investigation day of 23.07 μg/mm^3^ (for B-F00) and 25.94 μg/mm^3^ (for B-F02), respectively.

#### 3.1.3. Statistical Analyses of Water Sorption and Solubility

Two statistical tests were performed, the ANOVA OneWay to determine if there are any overall statistically significant differences between the materials, and the Tukey test for the post hoc daily comparison to determine where exactly the significant differences were noticed between the samples (*p* < 0.05). Regarding sorption, the Tukey test analyses indicate significant differences between all four sample groups, with the exception of the B-F02 and B-F03 pair. Regarding solubility, the Tukey test showed significant differences between all sample groups (*p* < 0.05).

### 3.2. Microstructural Analysis of Giomers Surfaces by SEM and AFM

#### 3.2.1. Microstructural Analysis of Giomers Surfaces by SEM

**Figure 5:**
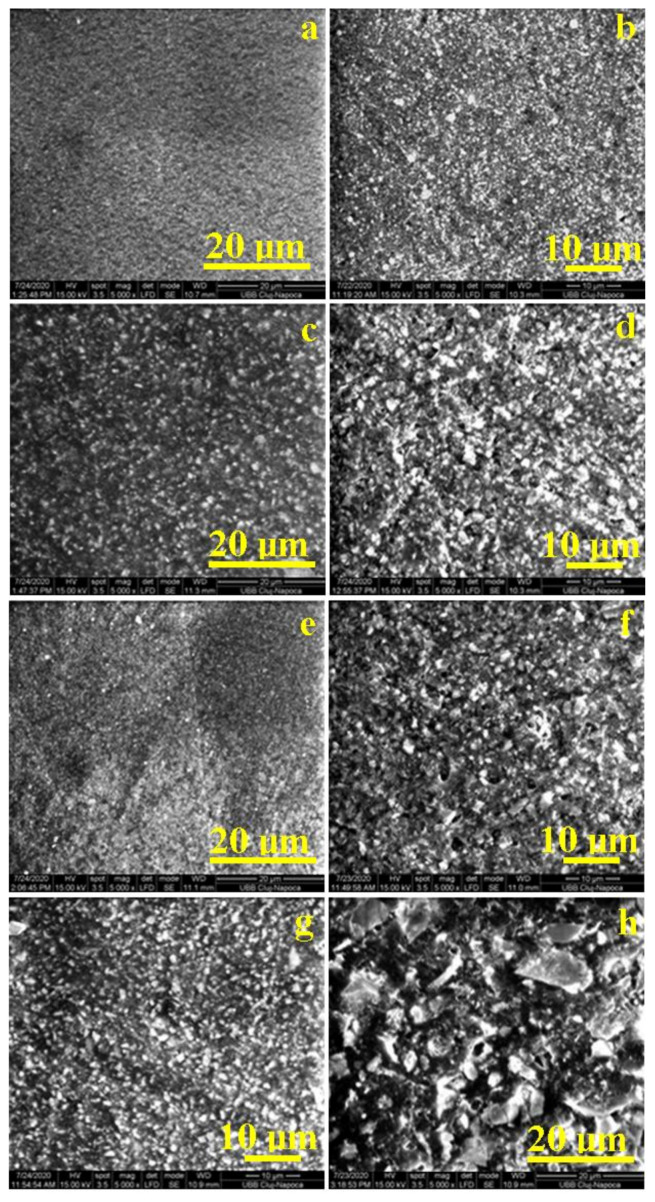
SEM images (×5000) of the surfaces of the investigated samples: (**a**,**b**) B-F00, (**c**,**d**) B-F02, (**e**,**f**) B-F03 and (**g**,**h**) G, before (left) and after (right) the 30 days of depositing in distilled water.

Figure 5 represents the SEM images of the samples before and after immersion in water.

Giomers B-F00 and B-F03 (Figure 5a,e) have similar morphological characteristics. Fine particles of filler evenly distributed in the matrix can be observed. The experimental giomer (Figure 5g) has a hybrid composition, with irregular particles. The surface morphology of G is similar to B-F02 (Figure 5c).

#### 3.2.2. Microstructural Analysis of Giomers Surfaces by AFM

In Table 2, the average roughness measured by AFM method, before and after the water treatment, is presented, alongside the standard deviation and the significance level (with *p* < 0.05 representing a statistically significant difference).

All materials show a significant increase in surface roughness after the wet exposure. Initially, B-F03 had the lowest surface roughness value, among the commercial samples and G the lowest surface roughness value among all analyzed samples. At the end of the wet exposure treatment, B-F00 presented the lowest surface roughness value among all analyzed samples. The most significant surface roughness increase was that of the experimental giomer G.

**Figure 6:**
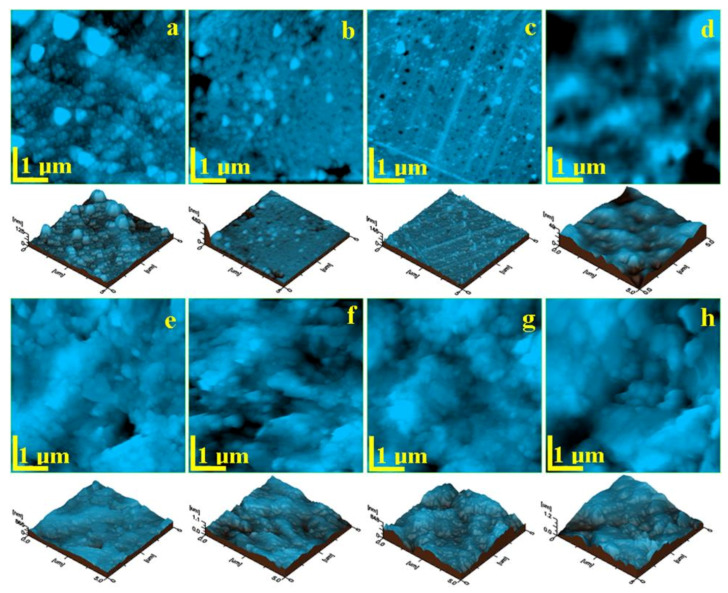
AFM topographic images of the initial samples: (**a**) B-F00, (**b**) B-F02, (**c**) B-F03, and (**d**) G; and samples after exposure: (**e**) B-F00, (**f**) B-F02, (**g**) B-F03, and (**h**) G. Scanned area 5 µm × 5µm. A three-dimensional view of the topography is given below of each image.

The initial samples of Beautifil materials have a similar surface topography evidencing the granular material being very well embedded into the organic matrix, Figure 6a–c. The notable difference among them is the diameter of the granular filler.

B-F00 presents large submicron particles spreading in a range of 100–600 nm surrounded by organic matter which bonds nanoparticles of about 60 nm, Figure 6a.

B-F02 has smaller submicron particles spread into the organic matrix having a diameter of about 300 nm. The nanoparticles of about 60 nm diameter are very well embedded into the organic material, Figure 6b.

B-F03 reveals the smoothest surface due to the predominant presence of the nanoparticles well embedded into the organic matrix instead of the submicron granular material. Only a few submicron granular particles are observed with a diameter in the range of 150–200 nm, Figure 6c. This influences the surface roughness which shows the lowest values for B-F03 among the Beautifil samples, Table 2.

## 4. Discussion

For all giomer materials, water interaction and the consecutive modifications are important for their efficiency as direct, esthetic and preventive restoration materials.

The null hypothesis of this study was rejected; the differences between the commercial and experimental giomers were statistically significant regarding the effects of immersion in liquid.

The hydrophilic nature of the matrix controls both the speed of diffusion and the water sorption degree [15]. However, in addition to the hydrophilic matrix, the giomers have a filling based on pre-reacted glass, and can be classified also according to the pre-reacted glass filler particles actively used in the chemical reactions, with the entire quantity being used up or with just the surface areas participating [16]. This signifies that the reacted glass-particles on the surface can become fluoride re-charging centers, but they can also lead to an accelerated water sorption and diffusion. Water sorption can be tolerated as long as it does not negatively impact the mechanical properties or if it does not lead to the over-inflation of the material that, in turn, leads to internal pressures in the restoration and, in the end, its failure. McCabe et al. [15] stipulated that, when compared to other materials, giomers tend to absorb more water—due to the osmotic effect generated by the presence of poly-acidic area inside the pre-reacted glass filler in the resin matrix. In another study, EL-Sharkawy et al. observed that water sorption has a significant influence on the color and marginal seal of the direct resin-based restoration [5,16,17]. At the same time, the functional particles, hydroxyl, carboxyl, and phosphate tend to bind to water by hydrogen bonds, further leading to inflating and plasticizing the polymer resin matrix [18,19].

All investigated materials in our study have a matrix containing Bis-GMA and TEGDMA. These polymers are hydrophilic and therefore will absorb water, leading to strong hydrogen bonds between the functional hydroxyl particle and water molecules. The smaller mass particle TEGDMA is highly fluidic, flexible and heterogeneous in its composition. Heterogeneity in the matrix allows larger micro-pores to be formed between polymers, leading to a significantly higher water sorption. These characteristics might explain the predisposition to absorb water that TEDGMA-based materials exhibit [5]. High water sorption values of giomer materials can be further explained by the acid–base reaction that takes place [20].

In the present study, the experimental giomer reached a value of 19.08 μg/mm^3^ after the first day of immersion in distilled water, 21.36 μg/mm^3^ after a week, and 22.44 μg/mm^3^ after 30 days. Material B-F03 had a water sorption of 15.467 μg/mm^3^ after the first day in the distilled water container, 19.248 μg/mm^3^ after one week, and a decrease after 30 days, reaching values close the ones registered on the first day, 15.409 μg/mm^3^.

For an earlier generation of flowable giomer, Beautifil Flow Plus F03, Harhash and colleagues registered values of 25.69 μg/mm^3^ after the first day of water sorption, 30.87 μg/mm^3^ after one week and after four weeks, 32.15 μg/mm^3^ [9,20]. In another study, after 84 days, the values reported for water sorption were 26.4 μg/mm^3^ for Beautifil Flow Plus F00 and for Beautifil Flow F02 45.9 μg/mm^3^ [20]. Sokolowski reported values of 26.4 μg/mm^3^ for Beautifil Flow Plus F00 and 45.9 μg/mm^3^ for Beautifil Flow F02 for water sorption, at the end of an 84-day evaluation [20].

Regarding the differences in material composition, it is worth noting that the filler percentage of Beautifil Flow Plus X F00 is 67.3 wt%, whereas for Beautifil Flow F02 it is 54.5 wt%. It was stated by Ferracane et al. that, the higher the filler percentage, the lower water sorption and solubility becomes, since it reduces the free volume of the resin matrix [21,22].

The experimental giomer G has a 60 wt% filling and after 30 days of water exposure, the sorption value was 22.44 μg/mm^3^. The closest value after 30 days was 17.00 μg/mm^3^ for B-F02 (54.05 wt% filler). In Figure 1, it can be observed that, after 30 days, the water sorption of investigated materials increased as follows: B-F00 < B-F03 < B-F02 < G.

In the present study, negative values were attained for water solubility of the materials. Other studies also reported negative values of the solubility parameter [23,24]. Some authors explained the negative values by the process of hydrolytic chemical reaction, leading to the formation of metal hydroxides on the surface of the filler particles [24]. Ortengren stated that the solubility of resin composites can be influenced both by the type of filler in the composition and also by the silane treatment applied [25]. Taking into account the possibility of incomplete dehydration of the materials, the negative values might only signify a low solubility level, rather than the absence of it. A possible explanation for the increase in the mass of giomer samples after dehydration (m3) would be the fact that a chemical reaction of the glass filler with water can take place inside the composite. After the water addition, the metal hydroxides can appear as reaction products on the surface of the filler particles. Another explanation would be that water molecules can form hydrogen bonds with the polar groups of polymer chains, and cannot be completely removed, a fact also supported by data from the literature [23]. Negative values might also be the result of hydrogen bonds between absorbed water molecules and functional polar particles of the polymeric chain, which cannot be completely eliminated [23]. In the literature, negative water solubility values of giomers [24,26] suggest that giomers are more susceptible to water sorption, and this phenomenon can hide the real solubility parameters. This is explained by the hydrophilic properties of the organic matrix.

Water sorption might also be influenced by the sample preparation procedure (mixing, working time, source of polymerization). Air bubbles, more common for flowable materials, could contribute to expanding the surface exposed to water contamination, inhibiting local polymerization, especially for materials with hydrophilic monomers in the matrix [21]. Studies show that the Beautifil Flow F02 (giomer) has a tendency to absorb a significant amount of water and, as a consequence, generates a strong osmotic effect [20]. The experimental giomer G exhibited similar values to the B-F02 giomer for the first investigation day. During the 2nd day, the sorption value of G was surpassed by B-F03. However, G displayed the highest overall sorption for the rest of the period, with B-F02 having the closest value to it.

Swelling can be detected in all materials, after a period of 30 days of water exposure, by examining the surface structure before and after this process. Multiple gaps were also observed, which come in accordance with the higher water sorption and solubility, when compared to materials that do not contain pre-reacted glass filler.

SEM imaging shows that the smoothest surface belongs to the giomer B-F00. This finding is also confirmed via AFM (Figure 6b). For B-F02 (Figure 6d), gaps and groves, where water eroded the material, are visible on the surface. In the case of the experimental giomer (Figure 5h), experimental pre-reacted glass filler particles are easily recognizable by the irregular shape and size. Small gaps and glass particle corners can be observed, piercing the surface of the resin matrix they are incorporated in. Higher values of water sorption for G and B-F02 (at 30 day) come in accordance with the SEM and AFM imaging performed after the sorption test. Regarding surface roughness and morphological characteristics through SEM imaging, G displayed most similarities with B-F02. However, at the end of the experiment, G had the roughest surface.

In AFM exposure, the experimental giomer also features a different topography than those of the Beautifil group. It contains a complex granular material very well mixed and embedded into the organic matrix that do not reach the top of the surface; Figure 6d. We can identify nanoparticles having a diameter of about 100 nm; submicron formations of about 600–800 nm in diameter and some micro-particles with a diameter of about 1.5 µm. The aspect of the granular material is blurred in the topographic image; Figure 6d, because of the position below the most superficial layer of the sample which only consists of organic material. This leads to the lowest surface roughness among of the initial samples, Table 2.

Distilled water was found to increase the surface roughness in relation to the water sorption and solubility process [5]. After 30 days of exposure, the samples are affected in a similar manner. The surface of the organic matrix is subjected to a continuous wet exposure which partially erodes the polymer. Therefore, the granular material becomes increasingly exposed at the surface. Some of the weaker bonded particles are washed away leading to local deformations, significantly enhancing the roughness, Table 2.

The topography after 30 days of exposure, Figure 6e–h, shows a rough surface formed by a compact and homogeneous mixture of granular material and organic matrix. It seems that the non-homogeneous filler areas were attacked and removed from the surface by wet exposure, while the homogeneous mixture could withstand the erosive effect.

The best result in terms of erosion resistance in the Beautifil group was observed for the sample B-F00 and the most eroded one was B-F02. The experimental giomer was the most affected, featuring greater roughness increase among all investigated samples. It is believed that this is due to the removal of all organic superficial layers until the compact structure was reached.

## 5. Conclusions

The experimental giomer G and the commercial material B-F02 have similar filler values, making them display a somewhat similar behavior when exposed to a wet environment.

All investigated materials have negative solubility values, probably due to the hydrogen bonds formed between absorbed water molecules and functional polar particles of the polymeric chain, which cannot be completely eliminated.

Regarding water sorption, the analyses showed significant differences between all four sample groups (except the B-F02 and B-F03 pair). The highest overall sorption values at the end of the investigation period were found for the experimental giomer G and B-F02, also visible on the SEM and AFM imaging performed after the water sorption test.

The experimental giomer G and the commercial material B-F02 have very close filler percentages, making them display a somewhat similar behavior regarding sorption and surface roughness when exposed to a wet environment. Similarities were also found between G and B-F03 regarding water solubility. However, the experimental giomer G will require further investigations and improvement in order to acquire optimal properties for dental applications.

## Figures and Tables

**Table 1:** Commercial Giomer composition (from manufacturers’ instructions—Shofu Dental Corporation, Japan).

Name	Consistency	Composition	Codification
Beautifil flow Plus X F00	Minimal flow	10–20%. Bis-GMA, TEGDMA, Bis-MPEPP, 50–60% S-PRG filler based on fluoroboroaluminosilicate glass, polymerization initiator, pigments and others	B-F00
Beautifil flow F02	Low flow	20–30%. Bis-GMA, TEGDMA, 40–50% S-PRG filler based on fluoroboroaluminosilicate glass, polymerization initiator, pigments and others	B-F02
Beautifil flow Plus X F03	Low flow	10–20%. Bis-GMA, TEGDMA, Bis-MPEPP, 50–60% S-PRG filler based on fluoroboroaluminosilicate glass, polymerization initiator, pigments and others	B-F03
*Experimental giomer	Flow	10–40%. *Bis-GMA, TEGDMA, 40–60% filler based on *Exp-glass, *SPRG, *FHAP, polymerization initiator, pigments and others	G

Bis-GMA (bisphenol A-glycidyl methacrylate), TEDGMA (triethylene glycol dimethacrylate), S-PRG (pre-reacted glass ionomer), Bis-MPEPP (polyethoxy dimethacrylate). *Bis-GMA, *SPRG, *Exp-Glass and *FHAP obtained at the Babeș-Bolyai University, Raluca Ripan Institute for Research in Chemistry, (Cluj-Napoca, Romania). TEGDMA, CQ, DMAEM of the experimental giomer were purchased from Sigma Aldrich Chemical Co.

**Table 2:** Average roughness measured by AFM and statistical analysis, initial and after 30 days of exposure in distilled water.

Samples	Ra Initial	Ra after 30 Days of Exposure (*p*-Value)	Rq Initial	Rq after 30 Days of Exposure (*p*-Value)
B-F00	11.88 ± 2.32	73.28 ± 18.27 (<0.05)	17.34 ± 5.11	91.70 ± 22.59 (<0.05)
B-F02	14.49 ± 8.32	139.60 ± 24.69 (<0.05)	20.77 ± 10.14	179.40 ± 31.35 (<0.05)
B-F03	9.96 ± 6.02	105.94 ± 14.74 (<0.05)	13.854 ± 7.25	132.80 ± 16.72 (<0.05)
G	5.16 ± 0.712	159.60 ± 34.10 (<0.05)	6.50 ± 0.84	202.40 ± 39.11 (<0.05)

*Ra (baseline surface roughness) represents the arithmetic average of the absolute values of the roughness profile ordinates; *Rq represents the root mean square average of height deviation taken from the mean image data plane.

## Data Availability

The data presented in this study are available on request from the corresponding author.
